# 4-(4-*tert*-Butyl­benz­yl)-1-neopentyl-1,2,4-triazolium bromide

**DOI:** 10.1107/S2414314625000926

**Published:** 2025-02-07

**Authors:** Daniel R. Albert, Michael Gau, Edward Rajaseelan

**Affiliations:** a Department of Chemistry Millersville University,Millersville PA 17551 USA; b Department of Chemistry, University of Pennsylvania, Philadelphia, PA 19104, USA; University of Aberdeen, United Kingdom

**Keywords:** crystal structure, triazolium salt, heterocyclic ionic compound

## Abstract

The title 1,2,4-triazolium salt, C_18_H_28_N_3_^+^·Br^−^, crystallizes in the monoclinic space group *Pc*. The extended structure exhibits a short inter­molecular inter­action between a heterocyclic C—H group and a bromide ion (H⋯Br = 2.57 Å). Additional weaker inter­actions exist between the other heterocyclic C—H group, an alkyl C—H group and bromide ions.

## Abstract 2

The title 1,2,4-triazolium salt, C_18_H_28_N_3_^+^·Br^−^, crystallizes in the monoclinic space group *Pc*. The extended structure exhibits a short inter­molecular inter­action between a heterocyclic C—H group and a bromide ion. Additional weaker inter­actions exist between the other heterocyclic C—H group, an alkyl C—H group and bromide ions.

## Graphical abstract

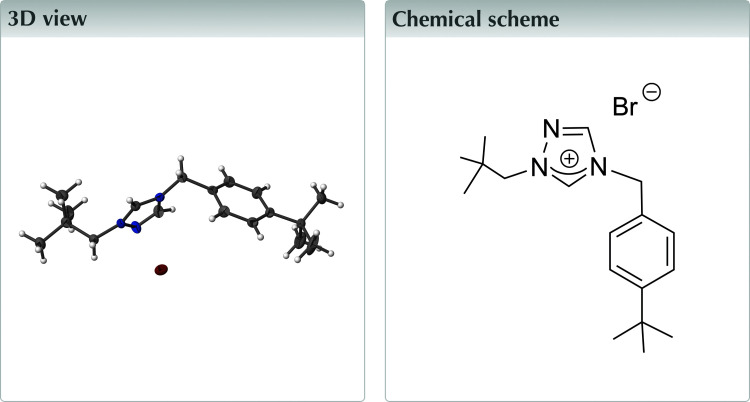

## Structure description

Asymmetric 1,2,4-triazolium cations are precursors for the synthesis of *N*-heterocyclic carbenes (NHCs) and are also of inter­est due to their utility as cations in ionic liquids (ILs) (Dwivedi *et al.*, 2014 ▸; Nelson, 2015 ▸; Strassner *et al.*, 2013 ▸; Riederer *et al.*, 2011 ▸; Chianese *et al.*, 2004 ▸). The crystal structures of several triazolium salts have been reported (Peña Hueso *et al.*, 2022 ▸; Kumasaki *et al.*, 2021 ▸; Ponjan *et al.*, 2020 ▸; Guino-o *et al.*, 2015 ▸; Maynard *et al.*, 2023 ▸). We have synthesized many imidazolium and triazolium salts as precursors in the synthesis of NHC complexes of rhodium and iridium (Castaldi *et al.*, 2021 ▸; Gnanamgari *et al.*, 2007 ▸; Idrees *et al.*, 2017 ▸; Lerch *et al.*, 2024 ▸; Nichol *et al.*, 2011 ▸; Newman *et al.*, 2021 ▸; Rushlow *et al.*, 2022 ▸).

**Figure 1:**
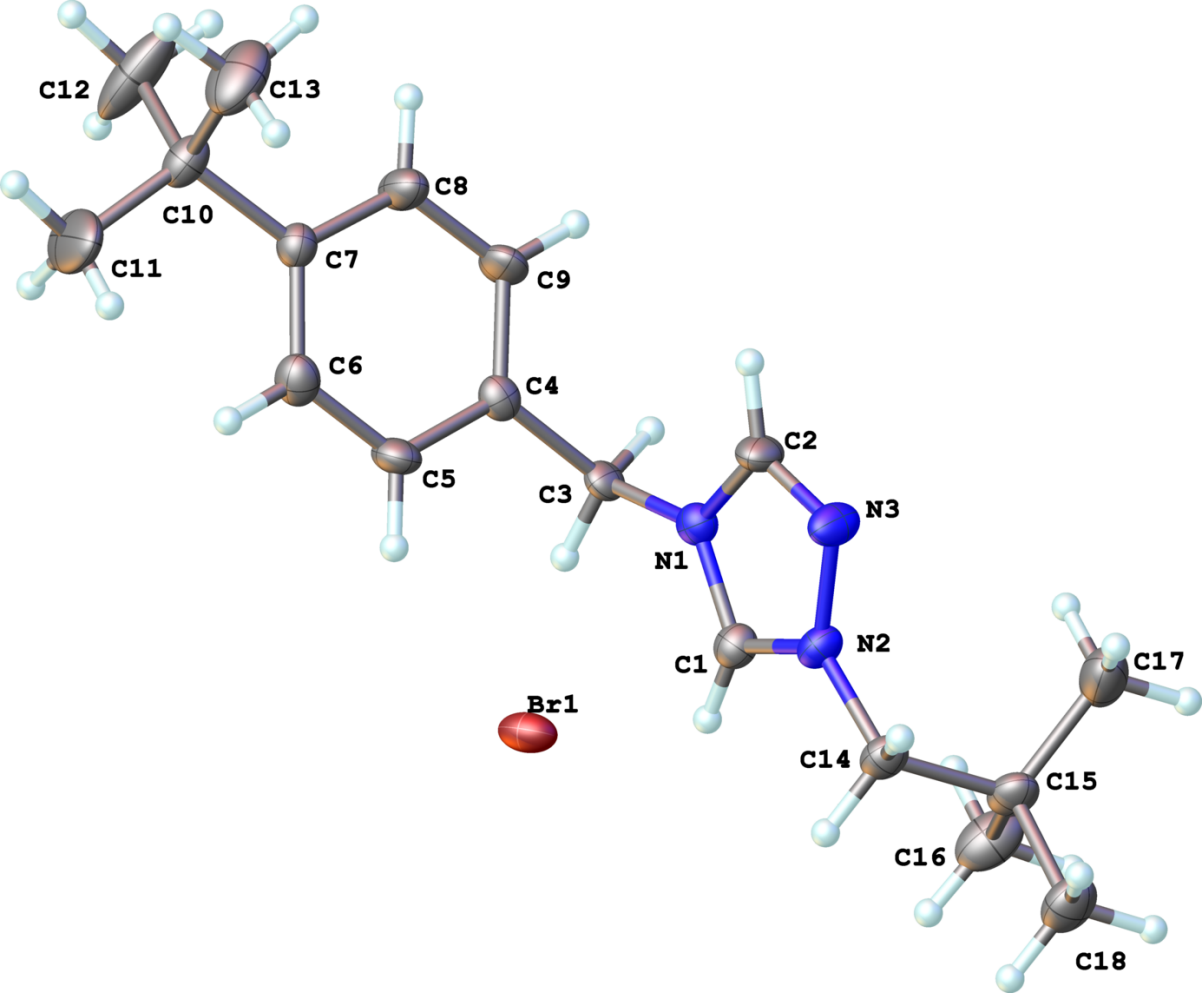
The mol­ecular structure of **2** with displacement ellipsoids drawn at the 50% probability level.

The mol­ecular structure of the title complex, C_18_H_28_N_3_^+^·Br^−^, **2** (Fig. 1 ▸), consists of a triazolium cation and a bromide counter-ion. The bond lengths in the triazolium ring indicate aromaticity with C—N bonds exhibiting distances in the range of 1.292 (9)–1.368 (9) Å and an N—N bond distance of 1.376 (8) Å; the N—C—N bond angles range from 107.9 (5) to 112.1 (6)°. The bulky neopentyl and 4-tertbutyl benzyl substituents on the nitro­gen atoms are in the expected *anti*-conformation with respect to the triazolium ring.

**Figure 2:**
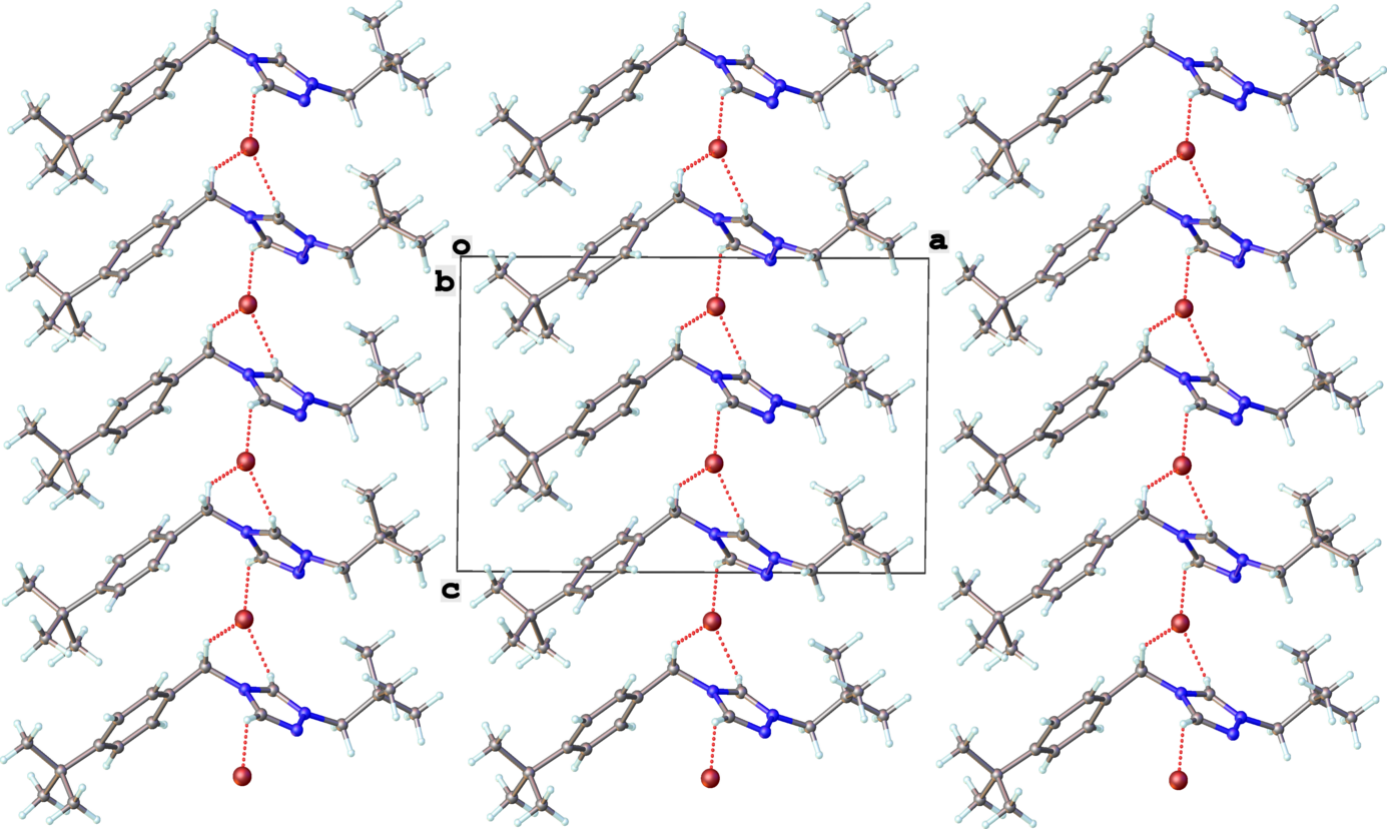
Crystal packing of **2** viewed along the *b-*axis direction. C—H⋯Br non-classical hydrogen-bonding inter­actions are shown as dotted red lines.

In the extended structure of **2**, several C—H⋯Br^−^ inter­actions are observed for heterocyclic C—H groups and an alkyl C—H group (Table 1 ▸). The non-classical hydrogen-bonding inter­actions are shown as dotted red lines in Fig. 2 ▸. The shortest non-standard hydrogen-bonding inter­action occurs between the most acidic hydrogen atom (C1—H1) and the bromide anion.

## Synthesis and crystallization

**Figure 3:**
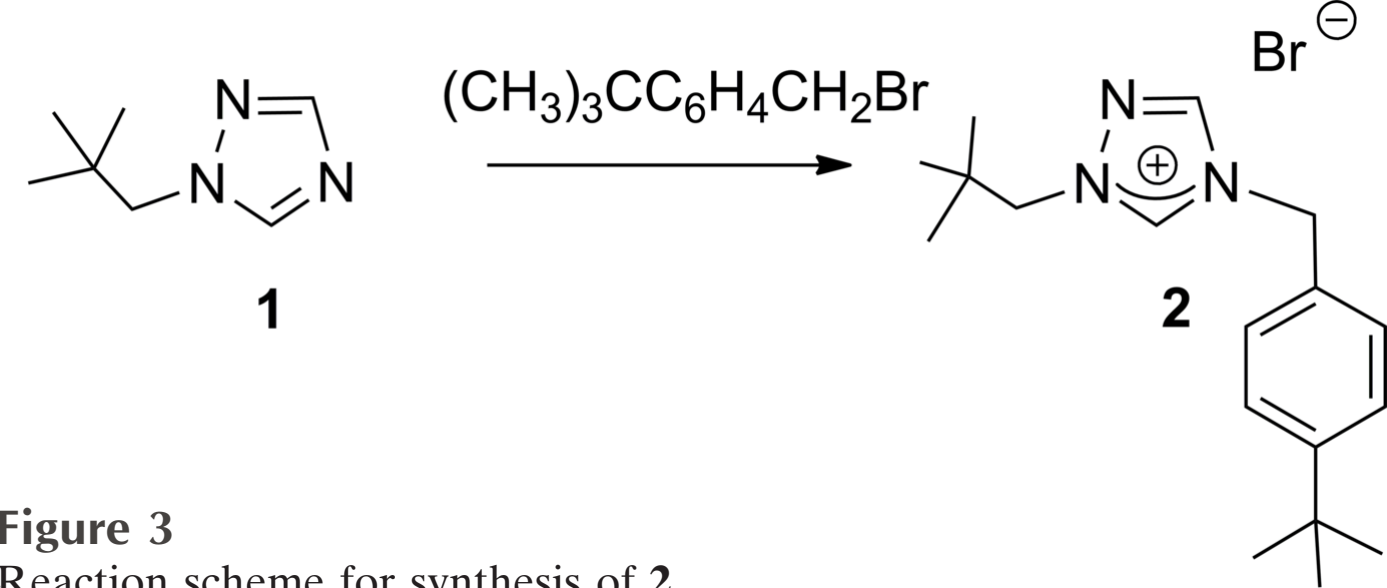
Reaction scheme for synthesis of **2**.

1-Neopentyl triazole (**1**) was synthesized as previously described (Mata *et al.*, 2003 ▸). All other compounds used in the syntheses as shown in Fig. 3 ▸ were obtained from Sigma-Aldrich and used as received. The synthesis was performed under nitro­gen using reagent-grade solvents, which were used as received without further purification. NMR spectra were recorded at room temperature in CDCl_3_ on a 400 MHz Varian spectrometer and referenced to the residual solvent peak (δ in p.p.m.). The title compound (**2**) crystallized as colorless needles by slow diffusion of pentane into a CH_2_Cl_2_ solution.

**1-Meopentyl-4-(4-*****tert*****-butyl­benz­yl)-1,2,4-triazolium bro­mide (2)**: 1-neopentyl-1,2,4-triazole (**1**) (1.67 g, 11.98 mmol) and 4-*tert*-butyl­benzyl bromide (5.34 g, 23.49 mmol) were added to degassed toluene (20 ml) and the mixture was refluxed in the dark for 72 h. After cooling, ether (75 ml) was added and the white solid that formed was filtered, washed with ether and air dried. Yield: 3.26 g (74%). ^1^H NMR: CDCl_3_, δ (p.p.m.) 11.92 (*s*, 1 H, N—C_5_H—N), 8.62 (*s*, 1 H, N—C_3_H—N), 7.55 (*d*, 2H, H_arom_), 7.44 (*d*, 2H, H_arom_), 5.84 [*s*, 2H, N—CH_2_ of CH_2_C_6_H_4_C(CH_3_)_3_], 4.28 [*s*, 2 H, CH_2_ of CH_2_C(CH_3_)_3_], 1.29 [*s*, 9 H, CH_3_ of C_6_H_4_C(CH_3_)_3_], 1.04 [*s*, 9 H, CH_3_ of CH_2_C(CH_3_)_3_]. ^13^C NMR: δ 153.26 [C_arom_ of C—C(CH_3_)_3_], 143.5 (N—C_3_H—N), 142.61 (N—C_5_H—N), 129.09, 128.82, 126.70 (C_arom_), 63.64 [N—CH_2_ of CH_2_C(CH_3_)_3_], 51.87 [N—CH_2_ of CH_2_C_6_H_4_C(CH_3_)_3_], 34.78 [C of C_6_H_4_C(CH_3_)_3_], 32.68 [C of CH_2_C(CH_3_)_3_], 31.17 [CH_3_ of C_6_H_4_C(CH_3_)_3_], 27.27 [CH_3_ of CH_2_C(CH_3_)_3_].

## Refinement

Crystal data, data collection, and structure refinement details are summarized in Table 2 ▸. The final model was refined as an inversion twin with a Flack parameter of 0.31 (4).

## Supplementary Material

Crystal structure: contains datablock(s) I. DOI: 10.1107/S2414314625000926/hb4505sup1.cif

Structure factors: contains datablock(s) I. DOI: 10.1107/S2414314625000926/hb4505Isup2.hkl

Supporting information file. DOI: 10.1107/S2414314625000926/hb4505Isup3.cml

CCDC reference: 2420832

Additional supporting information:  crystallographic information; 3D view; checkCIF report

## Figures and Tables

**Table 1:** Hydrogen-bond geometry (Å, °)

*D*—H⋯*A*	*D*—H	H⋯*A*	*D*⋯*A*	*D*—H⋯*A*
C1—H1⋯Br1^i^	0.95	2.57	3.446 (6)	154
C2—H2⋯Br1^ii^	0.95	2.75	3.550 (6)	143
C3—H3*B*⋯Br1^iii^	0.99	2.78	3.599 (7)	141

Symmetry codes: (i) 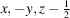; (ii) 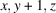; (iii) 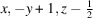.

**Table 2:** Experimental details

Crystal data	
Chemical formula	C_18_H_28_N_3_^+^·Br^−^
*M*_r_	366.34
Crystal system, space group	Monoclinic, *Pc*
Temperature (K)	100
*a*, *b*, *c* (Å)	14.9741 (2), 6.3842 (1), 10.0741 (1)
β (°)	90.435 (1)
*V* (Å^3^)	963.03 (2)
*Z*	2
Radiation type	Cu *K*α
μ (mm^−1^)	2.90
Crystal size (mm)	0.32 × 0.06 × 0.01

Data collection	
Diffractometer	Rigaku XtaLAB Synergy-S
Absorption correction	Multi-scan [SCALE3 ABSPACK in *CrysAlis PRO* (Rigaku OD, 2024 ▸)]
*T*_min_, *T*_max_	0.676, 1.000
No. of measured, independent and observed [*I* > 2σ(*I*)] reflections	14617, 3683, 3564
*R*_int_	0.067
(sin θ/λ)_max_ (Å^−1^)	0.625

Refinement	
*R*[*F*^2^ > 2σ(*F*^2^)], *wR*(*F*^2^), *S*	0.043, 0.135, 1.15
No. of reflections	3683
No. of parameters	206
No. of restraints	2
H-atom treatment	H-atom parameters constrained
Δρ_max_, Δρ_min_ (e Å^−3^)	0.77, −0.87
Absolute structure	Refined as an inversion twin
Absolute structure parameter	0.31 (4)

Computer programs: *CrysAlis PRO* (Rigaku OD, 2024 ▸), *SHELXT2018/2* (Sheldrick, 2015*a* ▸), *SHELXL2018/3* (Sheldrick, 2015*b* ▸), *OLEX2* (Dolomanov *et al.*, 2009 ▸) and *publCIF* (Westrip, 2010 ▸).

## full crystallographic data

### 4-(4-tert-Butylbenzyl)-1-neopentyl-1,2,4-triazolium bromide Crystal data

**Table:**

C_18_H_28_N_3_^+^·Br^−^	*F*(000) = 384
*M**_r_* = 366.34	*D*_x_ = 1.263 Mg m^−^^3^
Monoclinic, *P**c*	Cu *K*α radiation, λ = 1.54184 Å
*a* = 14.9741 (2) Å	Cell parameters from 11602 reflections
*b* = 6.3842 (1) Å	θ = 2.9–74.4°
*c* = 10.0741 (1) Å	µ = 2.90 mm^−^^1^
β = 90.435 (1)°	*T* = 100 K
*V* = 963.03 (2) Å^3^	Needle, colorless
*Z* = 2	0.32 × 0.06 × 0.01 mm

### 4-(4-tert-Butylbenzyl)-1-neopentyl-1,2,4-triazolium bromide Data collection

**Table:**

Rigaku XtaLAB Synergy-S diffractometer	3564 reflections with *I* > 2σ(*I*)
Detector resolution: 10.0000 pixels mm^-1^	*R*_int_ = 0.067
ω scans	θ_max_ = 74.5°, θ_min_ = 3.0°
Absorption correction: multi-scan [SCALE3 ABSPACK in CrysAlis PRO (Rigaku OD, 2024)]	*h* = −18→18
*T*_min_ = 0.676, *T*_max_ = 1.000	*k* = −7→6
14617 measured reflections	*l* = −12→12
3683 independent reflections	

### 4-(4-tert-Butylbenzyl)-1-neopentyl-1,2,4-triazolium bromide Refinement

**Table:**

Refinement on *F*^2^	Hydrogen site location: inferred from neighbouring sites
Least-squares matrix: full	H-atom parameters constrained
*R*[*F*^2^ > 2σ(*F*^2^)] = 0.043	*w* = 1/[σ^2^(*F*_o_^2^) + (0.0959*P*)^2^ + 0.2583*P*] where *P* = (*F*_o_^2^ + 2*F*_c_^2^)/3
*wR*(*F*^2^) = 0.135	(Δ/σ)_max_ < 0.001
*S* = 1.15	Δρ_max_ = 0.77 e Å^−^^3^
3683 reflections	Δρ_min_ = −0.87 e Å^−^^3^
206 parameters	Absolute structure: Refined as an inversion twin
2 restraints	Absolute structure parameter: 0.31 (4)
Primary atom site location: dual	

### 4-(4-tert-Butylbenzyl)-1-neopentyl-1,2,4-triazolium bromide Special details

**Table:**

Geometry. All esds (except the esd in the dihedral angle between two l.s. planes) are estimated using the full covariance matrix. The cell esds are taken into account individually in the estimation of esds in distances, angles and torsion angles; correlations between esds in cell parameters are only used when they are defined by crystal symmetry. An approximate (isotropic) treatment of cell esds is used for estimating esds involving l.s. planes.
Refinement. Refined as a 2-component inversion twin.

### 4-(4-tert-Butylbenzyl)-1-neopentyl-1,2,4-triazolium bromide Fractional atomic coordinates and isotropic or equivalent isotropic displacement parameters (Å^2^)

**Table:**

	*x*	*y*	*z*	*U*_iso_*/*U*_eq_	
Br1	0.54656 (4)	0.06909 (7)	0.65493 (5)	0.0291 (2)	
N1	0.5500 (4)	0.4637 (8)	0.3783 (5)	0.0207 (10)	
N2	0.6783 (3)	0.3713 (9)	0.4539 (5)	0.0223 (9)	
N3	0.6620 (4)	0.5666 (8)	0.5062 (7)	0.0274 (14)	
C1	0.6101 (3)	0.3093 (10)	0.3806 (6)	0.0227 (11)	
H1	0.604702	0.177837	0.337098	0.027*	
C2	0.5849 (4)	0.6176 (10)	0.4576 (7)	0.0265 (12)	
H2	0.555844	0.746944	0.474936	0.032*	
C3	0.4647 (5)	0.4590 (10)	0.3070 (7)	0.0202 (12)	
H3A	0.454105	0.316252	0.271962	0.024*	
H3B	0.467148	0.556569	0.230747	0.024*	
C4	0.3883 (4)	0.5206 (10)	0.3966 (6)	0.0212 (11)	
C5	0.3528 (4)	0.3741 (10)	0.4850 (7)	0.0259 (11)	
H5	0.378248	0.238097	0.491755	0.031*	
C6	0.2802 (4)	0.4284 (9)	0.5630 (7)	0.0274 (14)	
H6	0.256577	0.327384	0.622421	0.033*	
C7	0.2407 (4)	0.6264 (10)	0.5570 (6)	0.0222 (10)	
C8	0.2798 (4)	0.7725 (11)	0.4722 (8)	0.0307 (13)	
H8	0.256487	0.910800	0.468385	0.037*	
C9	0.3527 (4)	0.7196 (9)	0.3927 (7)	0.0288 (13)	
H9	0.377835	0.821754	0.335496	0.035*	
C10	0.1587 (4)	0.6849 (11)	0.6395 (6)	0.0271 (12)	
C11	0.1228 (7)	0.5004 (18)	0.7177 (13)	0.067 (3)	
H11A	0.171894	0.432843	0.766215	0.101*	
H11B	0.095357	0.399365	0.656521	0.101*	
H11C	0.077949	0.549817	0.780724	0.101*	
C12	0.0862 (5)	0.773 (2)	0.5505 (9)	0.070 (3)	
H12A	0.110278	0.890299	0.498836	0.105*	
H12B	0.036538	0.823367	0.604882	0.105*	
H12C	0.064673	0.663981	0.489943	0.105*	
C13	0.1868 (6)	0.8531 (18)	0.7397 (10)	0.057 (2)	
H13A	0.236811	0.801050	0.793659	0.085*	
H13B	0.136364	0.886337	0.797395	0.085*	
H13C	0.205292	0.979608	0.692129	0.085*	
C14	0.7580 (4)	0.2541 (10)	0.4900 (6)	0.0252 (11)	
H14A	0.742071	0.103995	0.496963	0.030*	
H14B	0.778492	0.300990	0.578763	0.030*	
C15	0.8356 (4)	0.2761 (11)	0.3927 (7)	0.0286 (13)	
C16	0.8101 (5)	0.1791 (14)	0.2603 (8)	0.0421 (17)	
H16A	0.792902	0.032522	0.273682	0.063*	
H16B	0.861192	0.185658	0.200193	0.063*	
H16C	0.759767	0.256349	0.221446	0.063*	
C17	0.8590 (5)	0.5087 (15)	0.3764 (10)	0.0461 (18)	
H17A	0.807805	0.583173	0.337805	0.069*	
H17B	0.910499	0.522528	0.317528	0.069*	
H17C	0.873678	0.568915	0.463317	0.069*	
C18	0.9139 (4)	0.1561 (13)	0.4534 (8)	0.0374 (15)	
H18A	0.928094	0.213916	0.541221	0.056*	
H18B	0.966108	0.169448	0.395882	0.056*	
H18C	0.897962	0.007942	0.462197	0.056*	

### 4-(4-tert-Butylbenzyl)-1-neopentyl-1,2,4-triazolium bromide Atomic displacement parameters (Å^2^)

**Table:**

	*U*^11^	*U*^22^	*U*^33^	*U*^12^	*U*^13^	*U*^23^
Br1	0.0425 (3)	0.0204 (3)	0.0244 (3)	0.0068 (3)	0.0046 (2)	0.0019 (3)
N1	0.022 (2)	0.021 (3)	0.019 (3)	0.0001 (19)	0.002 (2)	0.0024 (19)
N2	0.019 (2)	0.026 (2)	0.021 (2)	0.005 (2)	−0.0004 (17)	0.000 (2)
N3	0.023 (3)	0.025 (3)	0.034 (3)	0.0024 (18)	−0.007 (3)	−0.011 (2)
C1	0.022 (2)	0.025 (3)	0.021 (3)	0.001 (2)	0.0011 (19)	−0.001 (2)
C2	0.026 (3)	0.017 (2)	0.037 (3)	0.002 (3)	0.000 (2)	−0.007 (3)
C3	0.021 (3)	0.016 (2)	0.024 (3)	−0.003 (2)	−0.002 (2)	−0.004 (2)
C4	0.022 (2)	0.022 (3)	0.019 (3)	−0.003 (2)	−0.003 (2)	0.000 (2)
C5	0.032 (3)	0.015 (2)	0.031 (3)	0.003 (2)	0.003 (2)	0.000 (2)
C6	0.025 (3)	0.031 (4)	0.026 (3)	−0.002 (2)	−0.001 (2)	0.006 (2)
C7	0.020 (2)	0.024 (3)	0.023 (3)	−0.001 (2)	0.0019 (19)	0.000 (2)
C8	0.032 (3)	0.025 (3)	0.036 (4)	0.007 (2)	0.010 (3)	0.008 (3)
C9	0.032 (3)	0.019 (3)	0.036 (3)	0.004 (2)	0.004 (2)	0.012 (3)
C10	0.020 (2)	0.038 (3)	0.022 (3)	0.005 (2)	0.001 (2)	−0.001 (2)
C11	0.055 (5)	0.054 (5)	0.094 (8)	0.007 (4)	0.053 (5)	0.013 (6)
C12	0.033 (3)	0.141 (10)	0.037 (4)	0.038 (5)	0.006 (3)	0.017 (5)
C13	0.041 (4)	0.076 (6)	0.053 (5)	0.003 (4)	0.013 (3)	−0.025 (5)
C14	0.023 (2)	0.027 (3)	0.025 (3)	0.006 (2)	0.000 (2)	0.003 (2)
C15	0.027 (3)	0.028 (3)	0.031 (3)	0.009 (2)	0.001 (2)	0.008 (3)
C16	0.041 (3)	0.058 (5)	0.028 (4)	0.015 (3)	−0.001 (3)	−0.006 (3)
C17	0.027 (3)	0.046 (4)	0.065 (5)	0.002 (3)	0.007 (3)	0.015 (4)
C18	0.026 (3)	0.046 (4)	0.040 (4)	0.008 (3)	−0.002 (3)	0.006 (3)

### 4-(4-tert-Butylbenzyl)-1-neopentyl-1,2,4-triazolium bromide Geometric parameters (Å, º)

**Table:**

N1—C1	1.335 (7)	C10—C13	1.530 (11)
N1—C2	1.368 (9)	C11—H11A	0.9800
N1—C3	1.460 (8)	C11—H11B	0.9800
N2—N3	1.376 (8)	C11—H11C	0.9800
N2—C1	1.316 (8)	C12—H12A	0.9800
N2—C14	1.453 (7)	C12—H12B	0.9800
N3—C2	1.292 (9)	C12—H12C	0.9800
C1—H1	0.9500	C13—H13A	0.9800
C2—H2	0.9500	C13—H13B	0.9800
C3—H3A	0.9900	C13—H13C	0.9800
C3—H3B	0.9900	C14—H14A	0.9900
C3—C4	1.514 (9)	C14—H14B	0.9900
C4—C5	1.399 (9)	C14—C15	1.532 (8)
C4—C9	1.378 (8)	C15—C16	1.517 (11)
C5—H5	0.9500	C15—C17	1.535 (11)
C5—C6	1.391 (9)	C15—C18	1.525 (8)
C6—H6	0.9500	C16—H16A	0.9800
C6—C7	1.396 (9)	C16—H16B	0.9800
C7—C8	1.397 (9)	C16—H16C	0.9800
C7—C10	1.535 (7)	C17—H17A	0.9800
C8—H8	0.9500	C17—H17B	0.9800
C8—C9	1.400 (9)	C17—H17C	0.9800
C9—H9	0.9500	C18—H18A	0.9800
C10—C11	1.517 (12)	C18—H18B	0.9800
C10—C12	1.513 (10)	C18—H18C	0.9800
			
C1—N1—C2	105.4 (5)	H11A—C11—H11B	109.5
C1—N1—C3	125.5 (5)	H11A—C11—H11C	109.5
C2—N1—C3	129.1 (5)	H11B—C11—H11C	109.5
N3—N2—C14	121.3 (5)	C10—C12—H12A	109.5
C1—N2—N3	110.4 (5)	C10—C12—H12B	109.5
C1—N2—C14	128.1 (6)	C10—C12—H12C	109.5
C2—N3—N2	104.1 (5)	H12A—C12—H12B	109.5
N1—C1—H1	126.0	H12A—C12—H12C	109.5
N2—C1—N1	107.9 (5)	H12B—C12—H12C	109.5
N2—C1—H1	126.0	C10—C13—H13A	109.5
N1—C2—H2	123.9	C10—C13—H13B	109.5
N3—C2—N1	112.1 (6)	C10—C13—H13C	109.5
N3—C2—H2	123.9	H13A—C13—H13B	109.5
N1—C3—H3A	109.4	H13A—C13—H13C	109.5
N1—C3—H3B	109.4	H13B—C13—H13C	109.5
N1—C3—C4	111.3 (5)	N2—C14—H14A	108.6
H3A—C3—H3B	108.0	N2—C14—H14B	108.6
C4—C3—H3A	109.4	N2—C14—C15	114.8 (5)
C4—C3—H3B	109.4	H14A—C14—H14B	107.6
C5—C4—C3	119.9 (6)	C15—C14—H14A	108.6
C9—C4—C3	121.1 (6)	C15—C14—H14B	108.6
C9—C4—C5	119.1 (6)	C14—C15—C17	109.4 (6)
C4—C5—H5	120.2	C16—C15—C14	109.7 (6)
C6—C5—C4	119.7 (6)	C16—C15—C17	110.9 (7)
C6—C5—H5	120.2	C16—C15—C18	109.6 (6)
C5—C6—H6	118.9	C18—C15—C14	106.4 (5)
C5—C6—C7	122.3 (6)	C18—C15—C17	110.7 (6)
C7—C6—H6	118.9	C15—C16—H16A	109.5
C6—C7—C8	116.9 (5)	C15—C16—H16B	109.5
C6—C7—C10	122.5 (6)	C15—C16—H16C	109.5
C8—C7—C10	120.6 (6)	H16A—C16—H16B	109.5
C7—C8—H8	119.3	H16A—C16—H16C	109.5
C7—C8—C9	121.4 (6)	H16B—C16—H16C	109.5
C9—C8—H8	119.3	C15—C17—H17A	109.5
C4—C9—C8	120.6 (6)	C15—C17—H17B	109.5
C4—C9—H9	119.7	C15—C17—H17C	109.5
C8—C9—H9	119.7	H17A—C17—H17B	109.5
C11—C10—C7	112.3 (6)	H17A—C17—H17C	109.5
C11—C10—C13	107.4 (8)	H17B—C17—H17C	109.5
C12—C10—C7	110.1 (5)	C15—C18—H18A	109.5
C12—C10—C11	110.0 (8)	C15—C18—H18B	109.5
C12—C10—C13	108.8 (8)	C15—C18—H18C	109.5
C13—C10—C7	108.1 (5)	H18A—C18—H18B	109.5
C10—C11—H11A	109.5	H18A—C18—H18C	109.5
C10—C11—H11B	109.5	H18B—C18—H18C	109.5
C10—C11—H11C	109.5		
			
N1—C3—C4—C5	−79.6 (7)	C3—C4—C9—C8	177.5 (7)
N1—C3—C4—C9	100.4 (7)	C4—C5—C6—C7	−0.2 (10)
N2—N3—C2—N1	1.0 (8)	C5—C4—C9—C8	−2.5 (10)
N2—C14—C15—C16	−65.7 (7)	C5—C6—C7—C8	−2.5 (10)
N2—C14—C15—C17	56.2 (8)	C5—C6—C7—C10	178.0 (6)
N2—C14—C15—C18	175.8 (6)	C6—C7—C8—C9	2.8 (10)
N3—N2—C1—N1	2.6 (7)	C6—C7—C10—C11	−4.4 (10)
N3—N2—C14—C15	−96.1 (7)	C6—C7—C10—C12	−127.4 (8)
C1—N1—C2—N3	0.6 (8)	C6—C7—C10—C13	114.0 (8)
C1—N1—C3—C4	129.5 (6)	C7—C8—C9—C4	−0.3 (12)
C1—N2—N3—C2	−2.2 (8)	C8—C7—C10—C11	176.1 (8)
C1—N2—C14—C15	89.9 (8)	C8—C7—C10—C12	53.1 (10)
C2—N1—C1—N2	−1.9 (7)	C8—C7—C10—C13	−65.6 (8)
C2—N1—C3—C4	−48.7 (9)	C9—C4—C5—C6	2.7 (9)
C3—N1—C1—N2	179.5 (6)	C10—C7—C8—C9	−177.7 (6)
C3—N1—C2—N3	179.1 (7)	C14—N2—N3—C2	−177.2 (6)
C3—C4—C5—C6	−177.3 (6)	C14—N2—C1—N1	177.2 (6)

### 4-(4-tert-Butylbenzyl)-1-neopentyl-1,2,4-triazolium bromide Hydrogen-bond geometry (Å, º)

**Table:**

*D*—H···*A*	*D*—H	H···*A*	*D*···*A*	*D*—H···*A*
C1—H1···Br1^i^	0.95	2.57	3.446 (6)	154
C2—H2···Br1^ii^	0.95	2.75	3.550 (6)	143
C3—H3*B*···Br1^iii^	0.99	2.78	3.599 (7)	141

Symmetry codes: (i) *x*, −*y*, *z*−1/2; (ii) *x*, *y*+1, *z*; (iii) *x*, −*y*+1, *z*−1/2.
